# An exploration of social determinants of health amongst internally displaced persons in northern Uganda

**DOI:** 10.1186/1752-1505-3-10

**Published:** 2009-12-15

**Authors:** Bayard Roberts, Vicky Norah Odong, John Browne, Kaducu Felix Ocaka, Wenzel Geissler, Egbert Sondorp

**Affiliations:** 1 Department of Public Health and Policy, London School of Hygiene and Tropical Medicine, Keppel Street, WC1E 7HT, UK; 2 Gulu Health Study, Gulu University, PO Box 166, Gulu, Uganda; 3 Department of Epidemiology and Public Health, University College Cork, Ireland; 4 Faculty of Medicine, Gulu University, PO Box 166, Gulu, Uganda

## Abstract

Social determinants of health describe the conditions in which people are born, grow, live, work and age and their influence on health. These circumstances are shaped by the distribution of money, power and resources at global, national and local levels, which are themselves influenced by policy choices. Armed conflict and forced displacement are important influences on the social determinants of health. There is limited evidence on the social determinants of health of internally displaced persons (IDPs) who have been forced from their homes due to armed conflict but remain within the borders of their country. The aim of this study was to explore the social determinants of overall physical and mental health of IDPs, including the response strategies used by IDPs to support their health needs. Northern Uganda was chosen as a case-study, and 21 face-to-face semi-structured interviews with IDPs were conducted in fifteen IDP camps between November and December 2006.

The findings indicated a number of key social determinants. Experiencing traumatic events could cause "over thinking" which in turn could lead to "madness" and physical ailments. Respondents also attributed "over thinking" to the spirit (*cen*) of a killed person returning to disturb its killer. Other social determinants included overcrowding which affected physical health and contributed to an emotional sense of loss of freedom; and poverty and loss of land which affected physical health from lack of food and income, and mental health because of worry and uncertainty. Respondents also commented on how the conflict and displacement and led to changes in social and cultural norms such as increased "adultery", "defilement", and "thieving". Response strategies included a combination of biopsychosocial health services, traditional practices, religion, family and friends, and isolating.

This study supports work exploring the political, environmental, economic, and socio-cultural determinants of health of IDPs. Addressing these determinants is essential to fundamentally improving the overall physical and mental health of IDPs.

## Introduction

Social determinants of health describe the conditions in which people are born, grow, live, work and age and their influence on health. These circumstances are shaped by the distribution of money, power and resources at global, national and local levels, which are themselves influenced by policy choices [1]. Armed conflict and forced displacement are important influences on determinants of health [1,2]. In 2008, 42 million people globally were estimated to be forcibly displaced from their home areas as a result of armed conflict [3-6]. Approximately 26 million of these were 'internally displaced persons' (IDPs) who were displaced within the borders of their country of nationality. In contrast to refugees, who have crossed an international border, IDPs have often received limited support and protection under international law, from the United Nations, and from national governments [3,7]. Indeed, the national government may be responsible for the displacement itself and for human rights abuses and poor health of IDPs while supposedly under their protection [5,8]. As a result, extremely poor physical and mental health outcomes are consistently reported amongst IDPs [9-11].

There is limited evidence on the social determinants of health for IDPs. A number of studies have examined the factors influencing health outcomes of IDPs in quantitative terms [11-13]. In contrast, primary studies which aim to provide a qualitative understanding of factors influencing overall health outcomes from the perspective of IDPs are rare. One study by Astier Almedom of anxiety and mental distress amongst IDPs in Eritrea described how prolonged displacement, loss of livelihoods and assets, and uncertainty over the future influenced mental distress [14]. A study of health needs of IDP women and men in Colombia by Mogollón Pérez *et al *noted how respondents frequently referred to the economic causes of poor health, in particular the loss of livelihoods and difficulties in finding new work. Increased vulnerability was also noted because of civil crime and violence in their new places of settlement [15]. In a separate study by Mogollón Pérez *et al *of women IDPs in Colombia, economic hardship and environmental conditions were noted as the major factors influencing health. Behaviour changes resulting from the displacement were also noted, including the reproduction of violence in the home [16]. However, these few studies provide only limited evidence on the social determinants of health of IDPs and the response strategies used by IDPs to support their health needs.

The concept of social determinants of health was used for this study because it adopts a broad understanding of health and the many underlying and interconnected influences on health. These include the economic, political, social, and environmental characteristics and conditions which influence people's health throughout their lives, including armed conflict. The social determinants concept also incorporates approaches such as the social production of disease approach on the structural causes of inequality and subsequent influence on health, the psychosocial model on the influence of perception and experience of personal status on health, and ecosocial theory which seeks to integrate biological, ecological and social factors [17]. Although the influence of armed conflict on social determinants of health has been acknowledged in influential work on social determinants of health, there has been little specific data on armed conflict within this work [1]. This study seeks to contribute to the evidence-base on social determinants of health of conflict-affected populations.

The specific aim of this study was to explore the social determinants of overall physical and mental health of IDPs, including the response strategies used by IDPs to support their health needs. Northern Uganda was chosen as a case-study because of the huge number of IDPs in the region and an extremely weak evidence base on social determinants of health amongst the Ugandan IDPs (no published studies could be located specifically this issue). A brief description on the armed conflict and internal displacement in northern Uganda is now given.

### Internal displacement in northern Uganda

Since 1986, northern Uganda has witnessed a war between the Ugandan government and a series of rebel movements, principally the Lord's Resistance Army (LRA) led by Joseph Kony. The LRA have fought a low-level guerrilla war to try and overthrow President Museveni's government, and to rebuild the perceived Acholi nation and culture in northern Uganda. The LRA built upon previous North-South divisions which have marked Ugandan politics and society since independence and grievances amongst the Acholi people against President Museveni and his government [18,19]. The civilian population has suffered killings, assaults, sexual violence, and the abduction of children to become fighters, forced labourers, and sex slaves for the LRA [18,20,21]. The majority of the affected population are Acholi people from the districts of Gulu, Amuru, Kitgum and Pader, who worked on small land-holdings for subsistence and income before the conflict.

The war has been marked by forced population displacement because of attacks on civilians by the LRA, and the government ordering civilians to leave their homes and move to government established camps. The government justification for this forced displacement was to reportedly protect the civilians from the LRA and aid the army's counter-insurgency campaign against the LRA. The government-led displacement was scaled up in 1996, with civilians often subject to violence by the Ugandan army and given less than 48 hours to leave their villages and move into the camps [22]. IDPs could generally not travel from the camps to their homes or farmlands because of insecurity and travel restrictions imposed by the Ugandan army. The majority of people lived in the camps for between 5 and 10 years and some as long as 15 years [23].

Up to 2 million people were forced to leave their homes and live in IDP camps. In the three most conflict-affected districts of Gulu, Kitgum and Pader there were around 100 camps which ranged in size from around 1,000 to as many as 60,000 inhabitants [24]. The camps were characterised by chronic over-crowding, poor housing, water and sanitation. The camp residents were reliant on food aid from the World Food Programme and suffered high levels of poverty as they generally could not travel back to the villages and farm lands.

Although the camps were supposedly established for the protection of civilians, the camps were frequently attacked by the LRA, and IDPs reported poor security and high levels of violent and traumatic events when living in the camps [25,26]. Human rights abuses against camp residents by the UPDF have also been documented [20,25]. It has been observed that 'protection' by the government in northern Uganda 'has in fact been a cover for violation and mass humiliation' [8].

The health system in northern Uganda has been seriously damaged by the conflict, with access to health care impeded by insecurity, travel restrictions, impoverishment, and lack of medicines and supplies. Extremely high rates of mortality and physical and mental illness amongst IDPs have been recorded [24,26,27]. The chief humanitarian official of the United Nations, Jan Egeland, described the situation in northern Uganda as "the biggest neglected humanitarian emergency in the world" [28].

In August 2006, the government of Uganda and the LRA signed a Cessation of Hostilities Agreement. This has resulted in significant improvements in the security conditions over the last few years and large numbers of IDPs have now finally returned to their home villages. However, a final peace agreement was expected in April 2008 but has yet to be signed and there remain grievances amongst the Acholi over marginalisation and victimisation by the Ugandan government [29].

## Methods

This study followed a qualitative approach, with qualitative methods recognised as providing an important contribution to the study of social determinants of health [1]. Qualitative methods help provide respondent's own explanations and interpretations of factors affecting their health; explore issues to which quantitative methods are less suited such as cultural beliefs or individual explanations for influences on health; explore relationships between social determinants of health; help generate research questions which can then be investigated through quantitative methods; explore in-depth issues raised through previous quantitative studies; and elicit information in situations where quantitative methods may not be feasible (e.g. insecure environments).

An interview-based assessment method was used, with face-to-face semi-structured interviews conducted with IDPs were conducted between 14 November and 4 December 2006. This method allowed respondents to more freely express themselves about sensitive issues than may have been the case with other methods such as group discussions where lack of privacy and group consensus may have stifled individual expression.

The study defined social determinants based on the description given at the beginning of this paper [1]. The study followed a broad definition of health as 'a state of complete physical, mental and social well-being and not merely the absence of disease or infirmity' [30]. This use of overall physical and mental health was preferred to specific health conditions so as to incorporate the multi-dimensional nature of health and encourage respondents to use their own conceptions of their health. The interviewer referred to 'health', or 'physical health' or 'mental health' if prompted, rather than specific diseases or disorders to try and avoid leading or restricting responses, and to allow respondents to conceive health on their own terms.

A topic guide for the interviews was developed based upon the aim of the study, through analysis of relevant literature, and discussions with people knowledgeable of the situation in northern Uganda. The interviews addressed six main topics of: experience of leaving village and moving and living in the camp; how the displacement experience affected health; the challenges faced living in the camp and how they affect health; factors that have affected health and why they have affected health; understanding and conception of health; and types of coping and support for health. Questions within each of these topics were deliberately broad to try and elicit a range of respondent beliefs and experiences. For example, the main questions for the topic of factors affecting health were: 'in your opinion, what do you think are the things that have affected your health (for good and for bad)?'; 'how have these things affected your health?'; and 'why do you think these things have affected your health?'. Questions were used flexibly to suit the respondent's answers, and prompts were used if required, including referring to information given in previous answers by the respondent.

The interviews took place in 15 randomly selected IDP camps spread throughout the districts of Gulu and Amuru in northern Uganda. The camp populations ranged in size from 15,000 to 43,000, and the camps exhibited the acute overcrowding, poor hygiene, sanitation and living conditions characteristic of the camps in northern Uganda. Respondents in each camp were purposively selected from an already assembled group of randomly selected camp residents used for a separate study reported elsewhere [31]. One person from the assembled group of 40 camp residents was then purposively selected to ensure a cross-section of women, men and different adult age groups. No prior knowledge of potential participants was known and the purposive selection was based on visual selection for sex and approximate age. The respondents were all IDPs aged over 18 years. Twenty one interviews were conducted in total as preliminary analysis indicated that the data received from these interviews was sufficient to realise the study aim as common themes were reoccurring.

The interviews were all conducted in the Acholi language, and led by one of the authors (VN) who is fluent in Acholi and English. Another author (BR) was present during the interviews to respond to any queries raised during the interview but was not directly involved in the interview process (VN translated respondent queries to BR but very few queries were raised by respondents). The translation and transcription of the data was completed by VN. The English version was reviewed and clarifications sought for any elements that were unclear. VN belongs to the same ethnic group of the respondents (Acholi) and there was a potential risk of magnifying the suffering to highlight the situation of the Acholi in northern Uganda. Prior discussions were held to prevent this risk occurring. Cross-checks were also made of the original recordings with the English translation. The authors had no professional affiliations which may have influenced the collection or interpretation of the findings.

The analysis was conducted by BR, following an inductive approach to identify health determinants and health responses (based upon the respondents' conceptions of health). Thematic content analysis was used, and themes and sub-themes were developed to give coherent categories for the data. Guidance was sought from the authors from northern Uganda (VN and FK) to address any issues that were not clear and explore key terms (e.g. the frequent use of the term "over thinking"). The data was then coded based upon the themes and sub-themes. An iterative process was applied, with the themes and sub-themes revisited, altered and additional themes/sub-themes added during the coding process. The coding was then reviewed and adjusted accordingly. NVivo software was used for the analysis. The analysis included the frequency with which the themes and sub-themes arose relating to health determinants and health responses. This frequency analysis was a heuristic device to help guide the analysis and reliably represent the respondent opinions and it was not intended to have any statistical validity [32].

The interviews were all completely anonymous and confidential. They were conducted in the camp in which the respondent lived and in a private space (generally outdoors and away from people passing by) to maintain confidentiality given the potentially sensitive nature of some of the responses. An information sheet was read out to respondents prior to the interview and left with the respondents, and a consent form was completed for all respondents. The interviews were audio recorded and the recordings stored securely. Ethical approval for the study was provided by the Ugandan National Council for Science and Technology, Gulu University, and the London School of Hygiene and Tropical Medicine. As the interviews may have included responses on experience of painful and traumatic events, referral information for support on mental health was provided if requested.

## Results

Twenty one IDPs were interviewed, 12 were women and 9 were men. The respondents ranged in age from 18 to 60, with an average age of 32. All were from the Acholi tribe, 13 identified themselves as of the Catholic faith, and eight of Pentecostal faith. There was one refusal to be interviewed but no reason was given. A replacement respondent was found. The results for determinants and response strategies are presented below using categories derived from the inductive, thematic analysis.

### Determinants of health

#### Traumatic events

Respondents noted the health effects of violent and traumatic events experienced during the war and displacement. The specific traumatic events mentioned commonly included abductions, killing, torture, atrocities and rape. Seventeen out of 21 respondents noted how these traumatic events caused "over thinking" (multiple thoughts, feeling that the mind isn't working properly, obsessive thinking). "Over thinking" appears to stand on the pathway from traumatic experiences and suffering to poor mental health and ultimately to severe mental disorders ("madness"). One man in Adak camp noted how:

"the conflict is also bringing mental illness as a result of bad acts. For example you can sit only to be told that a relative of yours has been killed. This could be your child whom you love most. Then you can think too much and this leads to mental illness".

Respondents also noted the effect on the mental health of people who may have been forced to commit the atrocities. A man from Amuru camp noted that "people were abducted and taken to the bush. They did a lot of dirty things and even killed so they came back when their brains were not OK".

Seven respondents linked the violence and killings in the war and resulting "over thinking" with traditional beliefs for the causes of poor health, particularly mental health. According to Acholi beliefs, the spirit (*cen*) of a killed person might return to disturb its killer, and respondents noted that killing people could result in "ghosts", "charms" and the "spirit of the dead" which bring on "madness". A 35 year old man in Ongako camp noted how "the blood [*remo*] of the people killed will lead to mental disorder of the person who killed. This has always been a belief in our culture".

Four respondents noted how "over thinking" from traumatic events reduced physical strength and energy. A woman in Omel Lapem camp noted how "the anger of losing all the children is breaking my energy all the more. I no longer have any energy of going to work". One respondent noted that "when I start thinking hard, it brings me body pain".

Respondents also commented on the importance of ending the violence, and how "if the war ends, the over thinking that causes madness and the bad killings that result into madness will not be there". However, this was tempered by concern over an enforced return process by the government without adequate security and protection. A woman in Anaka camp noted:

"They talk of taking people back home. If they take us without soldiers won't Kony [the LRA's leader] sweep all our children away?...When I know death is waiting, abductions is also waiting, there is war, I cannot have any happiness only sadness...I don't have any peace because there is still war".

#### Overcrowding

Almost all respondents commented on the overcrowded nature of the camps and this led to disease and poor physical health. Most frequently cited characteristics were "overcrowding", being "packed" and "packing together", "dirty water", "dirty places", and "dirtiness". These characteristics caused general "sickness", "cholera", and "measles". A man in Amuru camp noted how diarrhoea "can spread very quickly and it has killed very many because people are too packed". A woman from Te-Tugu Camp felt that:

"People should be taken back home so that there is fresh air because houses are spaced. People are too packed here. Somebody coughs from one corner, another from the other corner, and there is increased sickness".

Seven respondents expressed the problems of overcrowding with an emotional feeling of lacking freedom. One respondent noted how "the houses are very packed. You can't get freedom". Another respondent noted how "people should be allowed to return to their villages, be free and enjoy fresh air". A respondent from Ongako camp felt that "in the village there was no over thinking but now people are gathered together so this brings headache, over thinking and mental disorder".

#### Poverty

17 respondents commented on how the war and displacement to the camps had increased poverty and how this had affected their emotional and mental health. This was commonly expressed by participants in terms of lack of money or income, lack of housing, and ability to adequately provide for their children. A 50 year old man from Omel Lopem camp noted:

"Mental disorders can come as a result of too many thoughts about poverty. What you used to do and things you used to see and have are not there. If you think deeply about them, you can develop mental illness".

Seven respondents noted how their mental health was affected because the impoverishment meant they could no longer meet the needs of their children. A woman from Bobi camp said:

"Life in the camp is very difficult. What we can eat that can be enough for me and my children is not there. I cannot buy clothes for the children. It touches me so much in my brain and wants to develop mental illness in me".

The effect of poverty on the ability to care for children and payment of school fees was noted. A respondent from Amuru camp noted that "there is an old man who got mad. He tried to pay his son at school in vain due to lack of money...The old man got mad due to poverty and he could see his child not going to school".

Respondents viewed the lack of access to "lands" and "gardens" and having "nowhere to dig" as the main cause of impoverishment. This in turn affected mental health. A man in Bobi camp commented:

"Given this kind of life, over thinking about how you should live can also result to madness. There is nothing you can do to bring money to your land. You keep planning for what you cannot get. This can finally lead to madness".

A man from Opit camp noted that "I feel thoughts have filled my head. I have nowhere to dig. The money I used to get from home is all lost. I am now a poor man". The cyclical effect of poor health on ability to work was noted by respondents. One respondent in Anaka camp noted that "we are really over thinking. This affects my work".

Seven respondents commented on impoverishment affecting their physical energy and strength, causing weakness and bodily pain. A respondent noted how "here there is no means of getting money, food and nowhere to dig. The strength I had those days is no longer there". Three respondents noted the lack of money to travel to obtain medicines and access medical care. The combined effects of displacement, poverty and loss of vitality were noted by respondents. A 45 year old woman in Omel Lapem camp described how "I have now moved in two different camps, even if you were hard working you will get weak. I don't have any energy now".

Respondents again noted the importance of being allowed to return home for improving their health. A woman in Adak camp also felt:

"If this war ends, mental cases will be reduced and there will be general improvement on health. Because you go back home, you will stay there and resume your work as you used to do. This will reduce on madness because right now poverty can also lead to madness".

The war and displacement meant people could no longer access their lands and so had lost their source of food and income, and were reliant on food aid. Over half of respondents noted how the lack of food was causing physical health problems, particularly in terms of "energy" and "strength". One respondent from Ongako camp commented how "my energy has gone down because there is nothing I can eat to give me energy to do work... We shall never have strength to do work and we shall remain weak". Food insecurity was also linked with poor mental health by seven respondents. A 47 year old woman in Ongako camp noted the effect of impoverishment, lack of access to land and food upon her health:

"I feel sick all the time with pain. I cannot get food. That's how it is in the camp. There is no means of getting money, food and nowhere to dig. The strength I had those days is no longer there".

#### Changes in social norms

The effect of war, displacement, impoverishment and living conditions in the camps also led respondents to comment on the changes in social and cultural norms and standards, and how this was affecting their well-being. Respondents noted that living in the camps had increased activities such as "prostitution", "adultery", "defilement", and "thieving", particularly amongst young people and children. This caused "unhappiness" and "anger" amongst the respondents. One respondent noted how "we even share bedrooms with the children which bring shame". A 50 year old man from Omel Lopem camp noted that:

"Today you train your child, and the child of the neighbour comes and spoils it all. Formerly people lived on various different hill sides with different cultures and different teaching".

Five respondents noted alcohol consumption as contributing to poor mental health. Excessive drinking was viewed by some respondents as on the pathway between "over-thinking" and "madness". One respondent noted that "if you fail to control deep thinking and you resort to drunkenness, it can lead to mental illness".

### Response strategies

The response strategies used by IDPs to support their health needs are described below.

#### Biopsychosocial health services and traditional local practices

Nineteen of the respondents noted the use of biopsychosocial (biomedical and psychosocial) health services for their health needs. These services included health centres, hospitals, and health workers. Twelve respondents mentioned biopsychosocial services as sources of support for emotional or mental health problems, and three of these noted the value of community-based counsellors as a source of support before seeking help at the hospital.

Respondents also noted the use of traditional local practices as a response for physical and particularly mental health needs. These included the use of traditional healers, rituals, and traditional medicines. Five respondents noted the use of traditional healers, often in combination with biopsychosocial health services, for emotional or mental health needs. A man in Ongako camp noted that "I take the victim [of mental illness] to the hospital. If the hospital fails I will follow our culture and go to the witch doctor to find out the root cause of the problem". Respondents also noted the use of religion alongside traditional practices and biopsychosocial health services. A woman in Anaka camp preferred to "pray first...and then go to the witchdoctors to find out what has brought the madness. You can then finally try counsellors or health workers". A man from Pabbo camp felt that if someone is suffering from physical sickness, "you can go to a witch doctor. and also be saved in a born again church and God will help you if he so wish. You can also take the person to hospital and the treatment can reduce it.

#### Religion

Nine respondents noted the importance of Christian faith in consoling them and providing support, with specific activities including attending church, prayer and reading the bible. A woman in Amuru camp noted:

"I have a lot of anger but the reason I give up taking my life is when I go to the church and the bible consoles me. Then I give up as everything is worldly. We don't deal with hospital. We only depend on prayer".

However, a woman from Acet camp rejected religion, stating that "I have given up religion. It doesn't help me. After all, my children are all dead".

#### Family and friends

Ten of the respondents noted the value of seeking emotional support from family or friends. An 18 year old woman from Pawel camp noted:

"You go to friends and tell them and they advise you against thinking too much because it can result to sickness. I tell my husband that my brain is not working well or that I have pain which is giving me thoughts".

Three respondents noted the support provided simply by having company, rather than discussing specific painful issues. A 24 year old woman noted that "when I'm thinking too much I go to my friends and have other stories. I have never told anybody about the problem of over thinking that I have now". A woman in Amuru camp noted that "if you are over thinking and you try to involve somebody, the person may instead worsen it".

#### Isolating

Three respondents felt that not mixing with other people or "isolating" was their preferred response to emotional or mental health problems. These were all older women. A 45 year old woman from Te-Tugu Camp said "I isolate because of over thinking when I feel pain in my heart". A 60 year old woman from Acet camp noted that "when I have much thought, I isolate myself and sit in my house and sleep...I handle it alone".

## Discussion

Research on social determinants of overall health amongst forcibly displaced populations is at a nascent stage, particularly for IDPs. This study provides new evidence on social determinants of overall health of IDPs using evidence from northern Uganda. Work looking at specific mental health outcomes of conflict-affected populations has highlighted the influence of political, environmental, economic, and socio-cultural determinants and the underlying pervasive influence of conflict and displacement on these determinants [33-36]. The findings from this study in Uganda will now be discussed within these broader classifications of determinants, drawing on relevant literature on IDPs in northern Uganda and other forcibly displaced populations in low-income settings.

### Political determinants

IDPs are often subject to prolonged exposure to politically-induced collective violence as they often experience much greater insecurity than refugees. Much of the collective violence in northern Uganda has taken place against IDPs living in the camps [5,26]. The respondents were thus living in situations in which mental and physical distress was caused not only by past experiences but also the fear of continuing and future violence. This study provides new evidence on how IDPs in northern Uganda describe the effect of this violence on their mental health. Over three quarters of respondents discussed the effect of collective violence and traumatic events on "over thinking" which could lead to "madness". In the Acholi socio-cultural context "over thinking" can refer to the way in which stressful circumstances cause constant worrying, multiple or obsessive thoughts, difficulty in identifying a clear solution, and a feeling that the mind isn't working properly and is "stuck". The term relates to Acholi terms such as *par *and *two tam *which incorporate stress and depression-like symptoms and have been described in detail elsewhere [37]. Studies with other displaced populations have also noted use of the term "over thinking" as a description of poor mental health. Astier Almedom notes how Eritrean IDPs spoke of too much thinking [14]. Elizabeth Marie Coker noted in her study of Sudanese refugees in Cairo how respondents referred to "thinking too much" on their current conditions and past events which in turn "fuelled" their physical illness [38]. "Over thinking" was also referred to by study respondents as an outcome relating to other determinants, particularly economic determinants, and this is discussed further below.

"Madness" was attributed by some study respondents to the "spirits" and "ghosts" (*cen*) of people killed. The strong belief in *cen *is consistent with beliefs of other Luo-speaking peoples [39], but Harlacher *et al *note how in the context and aftermath of the war in northern Uganda, the vengeful ghosts of those who had died a violent death has become the most widespread interpretation of mental illness amongst those who committed violent acts or event witnessed such acts [40].

The study also provides new information on how the "over thinking" from political violence in northern Uganda has also affected IDPs physical health in terms of "energy", "strength" and "body pain". The connection between trauma and physical suffering was also observed in a study by Tina Sideris of Mozambican women refugees in which respondents reported how the traumatic effects of war and displacement caused physical deterioration, bodily distress, and loss of vitality [41].

### Environmental determinants

Environmental factors were noted by respondents, mostly in terms of "overcrowding" and "packing together" which resulted in disease and physical "sickness". This overcrowding was also equated with a sense of environmental "dirtiness" and a lack of "fresh air" and loss of "freedom" by study respondents. These findings reflects those of Elizabeth Marie Coker who noted that for Sudanese refugees, "breathing is directly related to physical constriction in that freedom of movement, freedom of cultural expression and physical space that one can call ones' own are critical to life, to being able to 'breathe' freely, to being human" [38]. The negative effects of overcrowding noted by respondents could also be related to having to share intimate social spaces, and settling with other clans, which Acholi and broader Luo-speaking peoples have strict beliefs about [42].

### Economic determinants

The economic impoverishment brought about by the war and displacement and the effect on health was highlighted by many respondents - again in terms of thinking too much which could lead to "madness". The mental health effects of not being able to provide for children were noted by a number of study respondents. This supports the observation by Ambrose Olaa of stress amongst Ugandan IDPs of trying to meet the costs of educating their children [43]; and also studies by Mogollón Pérez *et al *and Almedom on the stress of IDPs trying to ensure their children's education [14,16].

Study respondents noted that having "nowhere to dig" because of a lack of access to their land was a major cause of the poverty. This resulted in inadequate food which affected the "energy" and "strength" of respondents. Respondents noted the mental distress caused by the material losses of not being able to dig their land and get food and money from their crops. Ambrose Olaa believes the loss of livelihoods and dependency on food aid amongst IDPs in northern Uganda also led to a loss of self-esteem and feelings of inadequacy [43]. A study by Boutin and Nkurunziza noted how on Burundian IDPs felt the loss of livelihoods and reliance on food aid undermined their dignity. Sideris' study of women refugees from Mozambique noted how the separation from the land was identified by respondents caused a perceived loss of respect and injury to the "spirit" [41]. Other studies in Uganda have described the cultural importance of the land to the Acholi, with the anthropologist Sverker Finnström noting how "crops are central in the Acholi imagination of a good, healthy life" [44]. The cultural aspects of the land were not raised by respondents in this study, but further studies could explore how a sense of cultural loss from being separated from the land may influence health amongst forcibly displaced populations.

### Socio-cultural determinants

This study provides new evidence on the influence on health from the changing social behaviour and the erosion of Acholi social norms and values. Other studies in northern Uganda have also observed changing traditional social norms, changing behaviour of children, increased prostitution, and rising alcohol use amongst men [21,43,45]. Finnström notes how "young men and women complained that there is no guidance from more senior people, while older men and women saw few possibilities to guard and guide the youth" [44]. This perceived social change also relates to the overlapping influences of other determinants such as the impact of violence and resulting poor mental health which could influence individual behaviour; overcrowded living conditions which may have disrupted traditional cultural norms and behaviours; and impoverishment - particularly the lack of employment and the loss of farming lands and associated roles and functions.

This concern over changing social norms connects to broader concerns about the damage and destruction to Acholi culture caused by the political violence and displacement [18]. Halacher *et al *state that "the devastating impact of displacement on the social fabric of the communities can hardly be overestimated. A society once characterized by a high level of social cohesion and mutual support... is drifting towards a harsh and unbridled individualism. Tensions between young and old, male and female, and people in general...have been steadily increasing". They note how the loss of cultural values has led to fractured community coherence and the loss of mutual support, both of which are desperately needed in such times of suffering [40].

The link between social suffering and individual suffering has been documented with other forcibly displaced populations. Coker believes the Sudanese respondents "almost invariably saw social and emotional pain as leading to physical pain" [38]. Sideris notes how "the destruction of social and cultural order is manifested in subjective forms of distress reflecting the interdependence between psychological processes and social environments" [41].

### Responses

The importance of emotional and social support from family and friends was highlighted by study respondents, and this reflects findings from other studies with displaced populations [36]. Almedom who notes how social support and maintaining social cohesion amongst IDPs in Eritrea was seen as "very important because people who have shared the same experiences and suffered together tend to form strong support groups and provide safe space for expressions of pain, anger and grief" [14]. However, some respondents in this study preferred to "isolate" to help deal with "over thinking". In a study of returning abductees in northern Uganda, Joanne Corbin notes how avoidance of talking about traumatic events was a key contributor to psychological healing and this has also been noted in other studies of conflict-affected populations [38,46,47].

Nearly all respondents sought external support for physical and mental health problems. These included biopsychosocial sources such as hospitals, health centres, health workers and counsellors. They also include the use of traditional remedies and traditional healers, reflecting Acholi beliefs in the role of spirits, ghosts and charms as causes of poor health. While a health centre may be able to provide drugs, visiting the traditional healer was viewed as addressing the "root cause" of problem. Both of these sources of help were supplemented by prayer and religious support. This medical syncretism of health seeking practice and behaviour can be viewed as a response to the multiple causes attributed by respondents to poor health, including "spirits", "charms", and "ghosts" [48], and studies have promoted the importance of recognising this syncretism and cultural beliefs as part of psychosocial health interventions for conflict-affected populations [36,49,50].

The study findings on medical syncretism correspond with those by Harlacher *et al *who note the overlapping Acholi beliefs of "normal" disease which have their causes in the "natural" world, and "spirit-related" diseases which are attributed to the "supernatural". This belief in "spirit-related" diseases includes providing remedies and conducting ceremonies for people experiencing mental health problems arising from exposure to violent events and also from committing violent acts [40]. The importance of following traditional and religious ceremonies to help relieve trauma have been noted in a number of studies with displaced people [51-53]

Respondents in this study noted the value of traditional rituals and practices as a means of individual support, but they did not discuss it as a means community support. This contrasts with other studies from northern Uganda which observe the use of traditional rituals and practices as a means of reaffirming Acholi identity [40,43]. Corbin notes in her study of returning abductees in northern Uganda how rituals help to "reconnect the larger community's sense of share values, norms and history, and support the social reconstruction of communities" [47]. Finnström notes how spirit-related diseases affect not only individuals but also families and entire clans, and therefore the use of traditional responses helps not only individuals but also broader Acholi society [44]. Finnström notes how they can also represents a means of trying to exert control over a situation of apparent powerlessness brought about by the conflict and displacement [44]. Further studies could explore the link between traditional rituals and practices as a means of reaffirming cultural identity and improving individual health outcomes.

### Study limitations

This study has a number of limitations. First, the study was a short-term assessment based on single interviews, and a longer qualitative study using more qualitative methods would reveal more details and nuances. Second, the study had 21 participants and a larger sample may have yielded additional perspectives. However, the key themes described above were commonly re-occurring which suggested that a degree of saturation was reached. Third, it is possible that men may have felt less at ease with a female interviewer than a male one. However, there was no evidence of this given the openness of respondents, and preparatory discussions with local experts indicated this would not be a systematic problem. Fourth, the respondents were from an impoverished population suffering from prolonged conflict and dependence on aid, and there was a risk that they magnified accounts of their suffering in the hope of receiving aid even though it was clarified with the respondents prior to the interview that they would derive no material benefits from the study and that the study team were not from an aid organisation. Fifth, specific health conditions could also have been explored in-depth, rather than overall physical and mental health. However, the study deliberately wanted to address the multi-dimensional nature of health and encourage respondents to use their own conceptions of their health. Sixth, in order to gain respondent's explanations for how forced displacement had influenced their health, the interview questions were deliberately broad and did not mention specific determinants. This approach could have limited gaining a deeper understanding of these specific determinants and future studies could explicitly seek to investigate political, environmental, economic and socio-cultural determinants, the relationships between these determinants, and perceptions of the comparable severity of health problems attributed to these different determinants. Lastly, the focus of the interviews was on how the conflict and forced displacement had influenced health. This may have limited the range of responses to those focusing on the conflict and displacement, rather than attributing health outcomes to other non-conflict issues such as cultural change related to modernisation processes in Acholi society, political repression, or generalised poverty. The focus on conflict and forced displacement may also have encouraged respondents to reflect on their mental health rather than physical health. There had also been no recent communicable disease outbreaks and so physical health issues may possibly have been of less immediate concern to respondents.

## Conclusion

This study sought to increase the limited evidence-base on social determinants of health of IDPs by exploring how IDPs in northern Uganda account for their health in their own terms. It is hoped that this study helps draw attention to the experiences and conditions discussed by the respondents in this study, and encourages further research on the social determinants of health of people affected by armed conflict and forced displacement. The study provides evidence to indicate that fundamental political, economic, environmental and socio-cultural changes are required to improve the overall physical and mental health situation of IDPs. The provision of humanitarian aid and basic health services provided essential but limited, temporary relief. Adequate security is required to reduce future exposure to violence, and also reduce people's fear of future violence. It will also help ensure people's access to land and so support economic wellbeing. Response strategies should also be supported, recognising the medical syncretism and traditional coping practices in Acholi culture. A return home has finally taken place for the majority of IDPs, but a comprehensive peace settlement between the LRA and the Ugandan government has not been signed and there is no certainty of sustainable peace in northern Uganda. Without a meaningful peace and sufficient support for the safety and rehabilitation for IDPs in their home areas, the overall health of the people of northern Uganda may continue to be severely compromised.

## Abbreviations

IDP: internally displaced person.

## Competing interests

The authors declare that they have no competing interests.

## Authors' contributions

BR led the study concept and design, data analysis, drafting of the manuscript, and participated in the data collection. VN participated in the data collection and transcription, and review of the manuscript. JB participated in developing the study concept and design, and review of the manuscript. KFO, WG, ES participated in developing the study concept and design, and review of the manuscript. All authors read and approved the final manuscript.
